# *BRAF* V600E Inhibitor (Vemurafenib) for *BRAF* V600E Mutated Low Grade Gliomas

**DOI:** 10.3389/fonc.2018.00526

**Published:** 2018-11-14

**Authors:** Francesca Del Bufalo, Giulia Ceglie, Antonella Cacchione, Iside Alessi, Giovanna Stefania Colafati, Andrea Carai, Francesca Diomedi-Camassei, Emmanuel De Billy, Emanuele Agolini, Angela Mastronuzzi, Franco Locatelli

**Affiliations:** ^1^Department of Paediatric Haematology/Oncology, IRCCS Bambino Gesù Children's Hospital, Rome, Italy; ^2^Department of Imaging, Neuroradiology Unit, IRCCS Bambino Gesù Children's Hospital, Rome, Italy; ^3^Department of Neuroscience and Neurorehabilitation, Neurosurgery Unit, IRCCS Bambino Gesù Children's Hospital, Rome, Italy; ^4^Department of Laboratories, Pathology Unit, IRCCS Bambino Gesù Children's Hospital, Rome, Italy; ^5^Laboratory of Medical Genetics, IRCCS Bambino Gesù Children's Hospital, Rome, Italy; ^6^Department of Paediatric Sciences, University of Pavia, Pavia, Italy

**Keywords:** pediatric central nervous system tumors, low-grade gliomas, vemurafenib, targeted therapies, pediatric neuro-oncology

## Abstract

Low-grade gliomas (LGG) are the most common central nervous system tumors in children. Prognosis depends on complete surgical resection. For patients not amenable of gross total resection (GTR) new approaches are needed. The *BRAF* mutation V600E is critical for the pathogenesis of pediatric gliomas and specific inhibitors of the mutated protein, such as Vemurafenib, are available. We investigated the safety and efficacy of Vemurafenib as single agent in pediatric patients with V600E^+^ LGG. From November 2013 to May 2018, 7 patients have been treated in our Institution; treatment was well-tolerated, the main concern being dermatological toxicity. The best responses to treatment were: 1 complete response, 3 partial responses, 1 stable disease, only one patient progressed; in one patient, the follow-up is too short to establish the clinical response. Two patients discontinued treatment, and, in both cases, immediate progression of the disease was observed. In one case the treatment was discontinued due to toxicity, in the other one the previously assessed *BRAF* V600E mutation was not confirmed by further investigation. Two patients, after obtaining a response, progressed during treatment, suggesting the occurrence of resistance mechanisms. Clinical response, with improvement of the neurologic function, was observed in all patients a few weeks after the therapy was started. Despite the limitations inherent to a small and heterogeneous cohort, this experience, suggests that Vemurafenib represents a treatment option in pediatric patients affected by LGG and carrying *BRAF* mutation V600E.

## Introduction

Low-grade gliomas (LGG) are common tumors in children. The prognosis varies widely among the different tumor subgroups and is determined by several factors, including grading, location, age at diagnosis, and extent of surgery, the gross total resection (GTR) being one of the main factors affecting the chance of cure. Surgery, along with radio- and chemotherapy, is currently the standard of care in the treatment of these neoplasms. However, there is a subgroup of patients that is judged not amenable of GTR, mainly because of the localization and extent of the mass. Despite the low biological malignity of these tumors, patients with unresectable masses and with clinical progression of the disease undergo chemotherapy or radiotherapy, suffering for the short- and long-term toxicities associated with these regimens. The main approaches of conventional chemotherapy for LGG includes carboplatin and vincristine, TPCV (thioguanine, procarbazine, lomustine, and vincristine) and weekly vinblastine monotherapy (1). Bevacizumab is another promising approach as it has shown improvements in the treatment of optic pathway gliomas (2). All this considered, new approaches, tailored on the biological characteristics of the disease, are needed for these patients.

The main molecular alterations shown by LGG relate to the activation of the MAP Kinase (MAPK) pathway and can be caused by either duplication or mutation of the *BRAF* gene (3). Therefore, inhibitors of the MAPK pathway have been considered as a potential target of therapy for tumors harboring these types of alterations (4, 5). Vemurafenib is a competitive small molecule that selectively recognizes the ATP-binding domain of the *BRAF*^*V*600*E*^ mutant. It has proved effective in the treatment of metastatic melanoma, a neoplasm frequently mutated for *BRAF*. More recently, an activity of this drug was proved also in pediatric *BRAF*^V600E^ mutated malignant astrocytomas (6–8), while less data are available on the use of the drug in patients with LGG (9, 10). We herein present a retrospective, monocentric analysis of the safety and efficacy of Vemurafenib as single agent for the treatment of 7 patients affected by unresectable LGG.

## Materials and methods

We retrospectively evaluated the medical reports of patients diagnosed with LGG and treated with Vemurafenib at Bambino Gesù Children's Hospital in Rome. The histological diagnosis was obtained in all patients either from biopsy or resected part in those children undergoing partial resection. *BRAF* ^V600E^ mutation was assessed by immunohistochemistry in all patients and in one case, due to a progression of the disease under treatment, also through Sanger sequencing of the *BRAF* gene.

Exon 15 of BRAF, spanning the V600 locus, was amplified by PCR using the KAPA2G Fast HotStart PCR Kit (Kapa Biosystems) according to the manufacturer's protocol with the following primers: BRAF_Ex15_Fw: CTTCATAATGCTTGCTCTGATAG and BRAF_Ex15_Rv: CTAGTAACTCAGCAGCATCTCAG. The amplification product was purified and sequenced by using the BigDye Terminator Version 3.1 Cycle Sequencing Kit according to the manufacturer's protocol on a 3130XL automatic sequencer (Applied Biosystems).

**Figure 1:**
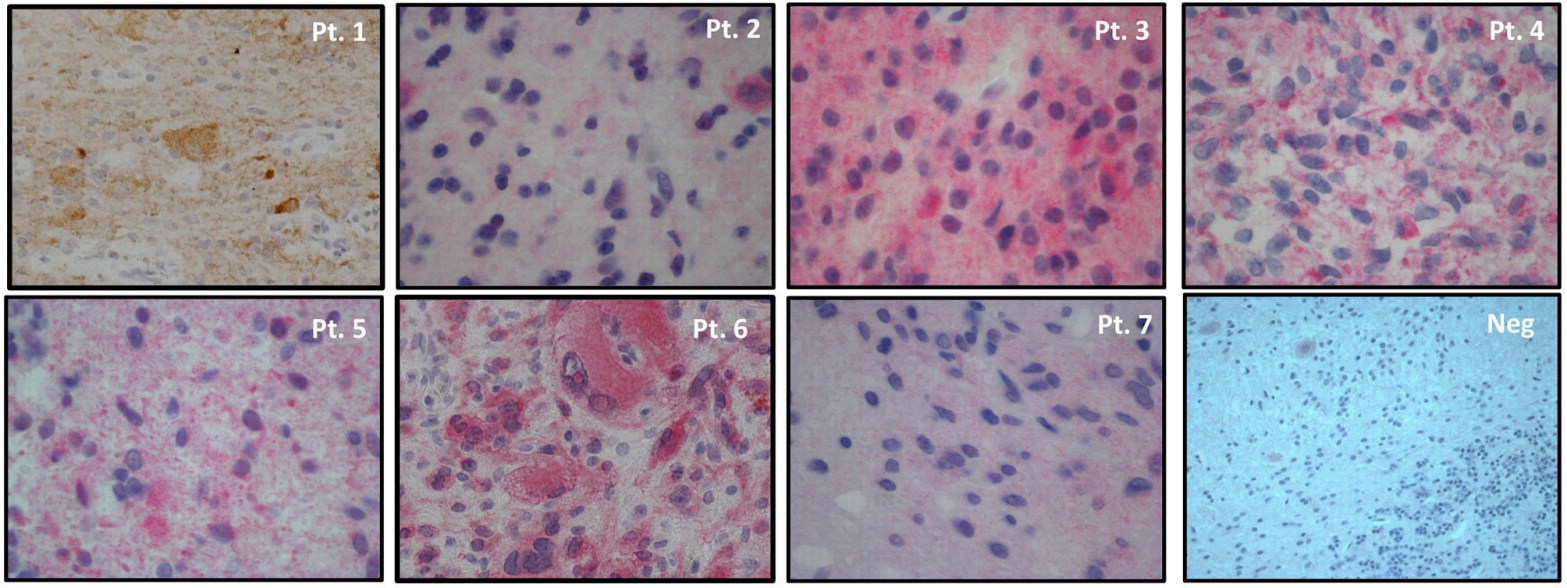
BRAF^V600E^ immunohistochemistry in tumor samples of patients' series and in normal cerebellar parenchyma (small neurons of cortical molecular layer, Purkinje cells and underlining white mater resulted negative). Magnifications: Pt. 1 20x, Pts. 2-7 40x, Neg. 20x. 3,3′-diaminobenzidine-tetrahydrochloride-dihydrate (DAB) substrate for Pt.1 and alkaline phosphatase substrate for Pts. 2-7 and negative control. Hematoxylin counterstain in all samples.

As for immunohistochemistry, 3-micron paraffin embedded sections were dehydrated, pretreated with avidin block for 15' and biotin block for 15', then incubated with BSA for 30' and incubated with mouse anti-human BRAF V600E monoclonal antibody (clone VE1, Spring Bioscience) at room temperature for 60' (dilution 1:50, PT-link antigen retrival at high pH). Incubation with biotinylated secondary antibodies for 15' at room temperature and incubation with alkaline phosphatase or 3,3′-diaminobenzidine-tetrahydrochloride-dihydrate (DAB) conjugated streptavidin for 15' at room temperature were performed. The BRAF staining for all patients is shown in Figure 1 and a quantitative measure of the intensity of the staining can be found in Table 1. Normal cerebellar parenchyma served as negative control (shown in the same Figure).

**Table 1:** Clinical-pathological characteristics of patients and BRAF^V600E^ immunostain intensity.

	**Pt 1**	**Pt 2**	**Pt 3**	**Pt 4**	**Pt 5**	**Pt 6**	**Pt 7**
Age (months)	28 m	54 m	1 m	108 m	89 m	122 m	125 m
Site	Cervico-medullary	Vermis + cerebellar hemisphere	Medulla oblongata	Midbrain	Medulla oblongata	Midbrain	Optic chiasm
Histology	GG	GG	Ganglioneurocytoma	PXA	GG	GG	Pylocitic Astrocytoma
BRAF^V600E^ IHC	++	++	+++	+++	+++	+++	+
Surgery	Biopsy	Partial resection	Partial resection	Partial resection	Partial resection	Partial resection	Partial resection
Previous CT	SIOP LGG 2004	No	No	No	No	No	No
Max dose	960 mg/day	480 mg/day	240 mg/day	960 mg/day	480 mg/day	480 mg/day	960 mg/day
Toxicity	Skin (grade 3)	No	No	Skin (grade 1)	Skin (grade 1)	Skin (grade 1)	Skin (grade 2)
Best response	PR	CR	PD	PR	SD	Insufficient follow-up	Treatment stopped for BRAF^V600E^ negativity at Sanger Sequencing
Follow-up/(months)	54 m	40 m	4 m	30 m	13 m	2 m	24 m
Duration of treatment (months)	54 m	40 m	3 m	30 m	13 m	2 m	9 m

*Pt 4 is also affected by constitutional chromosome 21 trisomy*.

*SIOP-LGG 2004: (Vincristine, Carboplatin, Etoposide). Acronyms: GG, ganglioglioma; PXA, pleomorphic xantoastrocytomas; IHC, immunohistochemistry; CT, Chemotherapy; PR, Partial Response; CR, Complete Response; PD, Progressive Disease; SD, Stable Disease*.

In the absence of pharmacokinetic and pharmacodynamic data of Vemurafenib in a pediatric population, we decided to start the treatment with the minimal dose proved to be active in adults (240 mg/day *per os* in 2 administrations). Subsequent dose adjustment was evaluated case by case, depending on drug tolerance. Radiologic response to treatment was evaluated and classified according to RECIST criteria; major responses included complete response (CR), partial response (PR) and stable disease (SD). Toxicity was graded according to the Common Terminology Criteria for Adverse Events (CTCAE), v 4.0. The mean time for the radiologic evaluation of response was 6 months for all the patients.

All the parents/legal guardians of patients provided formal, informed consent to the treatment and the study was approved by Institutional Review Board (Ospedale Pediatrico Bambino Gesù).

## Results

From November 2013 to May 2018, 7 patients have been treated; the main characteristics of the patients are reported in Table 1. The median age at diagnosis was 75.2 months (range 1–125); F:M ratio was 1:6. Histological diagnosis were: Gangliogliomas (GG) (4 patients), Pleomorphic Xantoastrocytoma (PXA, 1 patient), Ganglio-neurocytoma (1 patient) and Pylocitic Astrocytoma (PA, 1 patient). Only one of the seven patients had undergone previous treatment consisting of surgery and chemotherapy according to the treatment protocol SIOP LGG 2004 (including mainly Vincristine, Carboplatin, Etoposide).

Vemurafenib was started at a median time from diagnosis of 17 months (range 1–52) and was administered orally for a median of 22 months (range 3–52). Dose adjustments were carried out depending on observed toxicity and efficacy; in one case the dose remained 240 mg/day, in two cases was increased up to 480 mg/day and in four cases up to 960 mg/day. The median follow-up time from initiation with Vemurafenib therapy is currently 24 months (range 2–54).

The treatment was overall well-tolerated. Skin toxicity developed in 5/7 pts, reaching grade 3 (CTC v4.0) in one of them (at the dose of 960 mg/day) and leading to a 2-month discontinuation of the treatment that was later restarted without safety issues at the same dose. One patient developed grade 2 toxicity and the remaining 5 cases experienced only grade 1 toxicity, not requiring discontinuation of the treatment. The skin changes were mainly in the photo-exposed areas and consisted of a maculo-papular rash and diffused xerosis. These adverse effects occurred around 1 month after the treatment was started. In one case (the most severe one), the maximum grade of the lesions appeared after 15 months from the initiation of treatment, but shortly after (i.e., 1 month) the increase of the dose to 960 mg/day. The patients experiencing the highest toxicity (grade 2 and 4) were both receiving the maximum dose (960 mg/day). No skin tumor developed in any patient.

Although the cohort is too small and heterogeneous in terms of tumor histology, we observed 57% (4/7) major responses in our cohort. One child affected by GG obtained CR after 6 months and 2 patients (1 GG and 1 PXA) reached PR. Amongst these 2 latter patients, the one affected by PXA (who also had constitutional trisomy 21) had the best response to treatment after 3 months, while the other one showed the best response 12 months after the treatment was started, 2 months after initiating with the highest tolerated dose level of 960 mg/day. For the remaining 2 patients affected by GG, one remains in stable disease after 18 months of treatment and the other has a follow-up of 2 months only, therefore too short to evaluate the radiological response; he shows, however, a clinical response with improvement of the main neurological deficits (ataxia and visual impairment).The best radiological response was obtained 3 months after Vemurafenib was started and a mean sustained response of 2 years was observed. No correlation between *BRAF*^*V*600*E*^ positivity and treatment outcome was found.

Remarkably, a clinical response with improvement of the neurological function was observed early in all the responding patients, after 2 weeks of treatment.

The patient with the ganglio-neurocytoma progressed under treatment. This child had a bulky lesion involving the brainstem and cerebellum and presented with severe clinical conditions at diagnosis.

Lastly, in one patient affected by PA we observed disease progression under treatment with Vemurafenib. To understand the possible cause of this lack of response, we performed sequencing of the *BRAF* gene, which showed a negativity for the V600E mutation. The therapy was then interrupted, and the patient underwent partial resection with stabilization of the disease.

**Figure 2:**
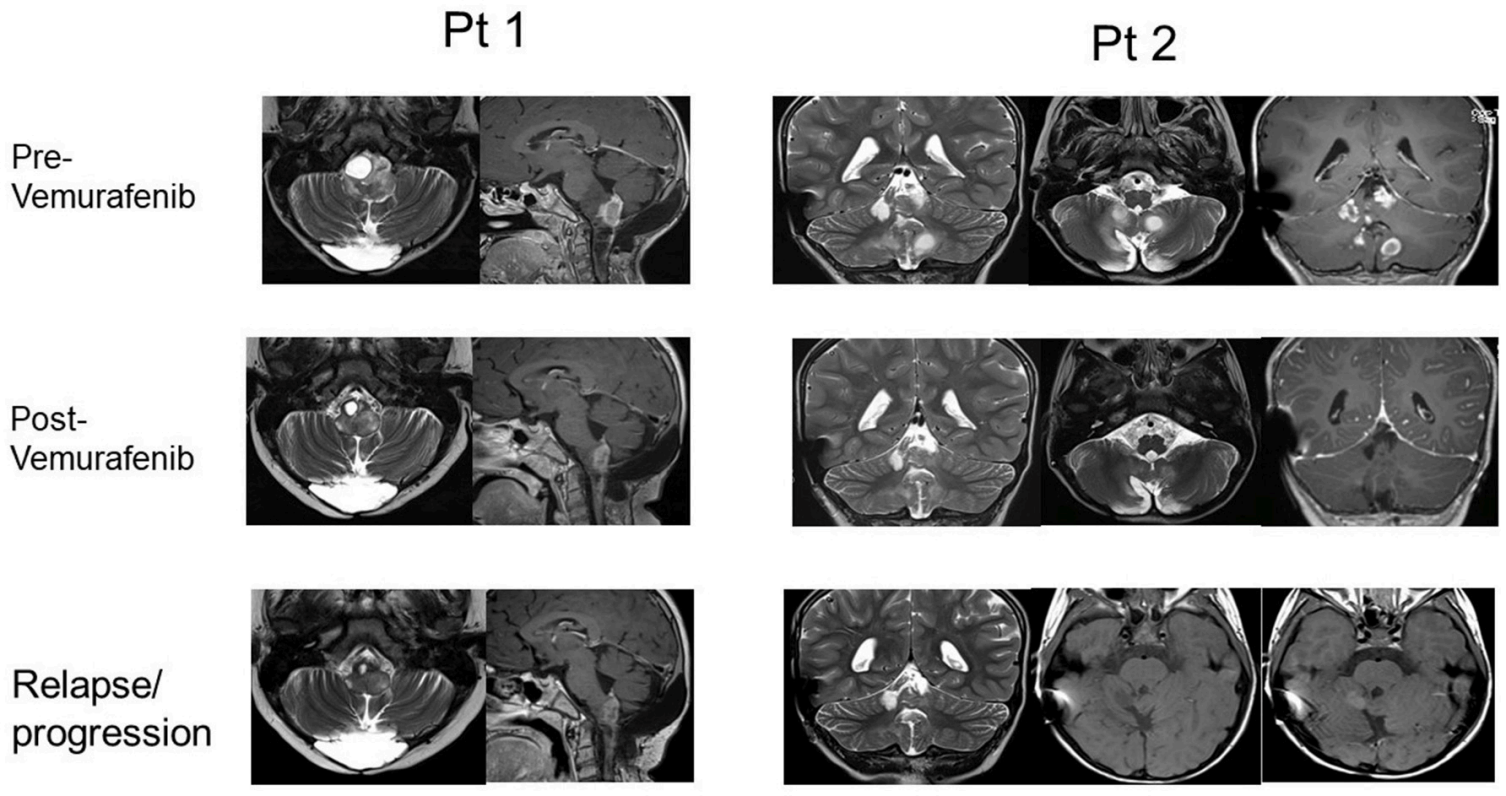
RMN imaging pre- and post- Vemurafenib (best response) and at relapse/progression after Vemurafenib of pts 1 and 2.

Moreover, two of the patients that showed a response (after obtaining PR in one case and CR in the other case), experienced tumor regrowth under treatment, 24 and 15 months after the start of the treatment respectively, suggesting the development of resistance (RM images in Figure 2). One case was treated with radiotherapy, obtaining a reduction of the mass and Vemurafenib was later reintroduced with a complete control of the disease. In the other case, the treatment was continued at the same dose, obtaining a new stabilization of the disease until the latest follow-up (40 months).

Not surprisingly, an immediate tumor re-growth was seen after suspension of the treatment for toxicity in one responder; the therapeutic benefit was, however, re-obtained with the resumption of the drug.

## Discussion

In the past few years, dramatic progresses have been made in the understanding of the biology of LGG, thanks to the advent of new methodologies in genomics. The identification of specific molecular signatures of these tumors is changing the treatment strategies and targeted therapies are currently being explored in clinical trials. In fact, the conventional treatment strategies for unresectable LGG include chemotherapy and radiotherapy, with the long term disabilities that these treatments determine. In view of the long term survival associated with these tumors, and especially when considering the pediatric age, reducing long-term toxicities is mandatory. Targeted agents have, by definition, less systemic and long term adverse effects, even if their relatively recent use does not enable a full characterization of these aspects yet.

One of the most notable findings in this ever-changing landscape includes abnormalities in the RAS/MAPK pathway, such as *BRAF* activation. The availability of specific inhibitors of the *BRAF*^V600E^ alteration such as Vemurafenib is an example of the possible therapeutic translation of these findings, paving the way for an innovative treatment option for a selected population of pediatric patients affected by LGG and harboring *BRAF*^V600E^.

We have presented the case of 7 patients with *BRAF* mutated LGG treated with Vemurafenib. This is, to the best of our knowledge, the first study to investigate safety and efficacy of this molecule in a pediatric population affected by LGG.

In all patients, the *BRAF* mutation was assessed through immunohistochemistry, and a further investigation through molecular assay was performed only in one case because of the poor response to the treatment observed, revealing a false positivity of the staining. Even though the gold standard method to assess BRAF status in patients with metastatic melanoma is based on molecular assays, the high costs and expertise required for the molecular assay is responsible for the limited use of the test. On the other side, the development of a mutation-specific monoclonal antibody (VE1), which enables the detection of the BRAF^V600E^ mutated protein by immunohistochemistry, made the analysis more widely available and less expensive, although less accurate as shown by our child with PA. In this regard, the possible inconsistency between these two methods (immunohistochemistry vs. molecular testing) is an issue that must be addressed. Immunohistochemistry may represent the best screening method in LGG patients, thanks to the wider availability and lower cost, but its lower specificity and sensibility when compared to molecular analysis must be always taken into account, particularly in those patients where the specific therapy does not seem to control the disease. In these cases, a molecular confirmation of the immunohistochemistry results is recommended.

The treatment appears to be well-tolerated, with only mild toxicity; the safety was confirmed also in a patient with constitutional trisomy of the chromosome 21. We observed exclusively skin toxicity, and only in one case the severity of the adverse effect led to the discontinuation of the drug. Dermatological toxicity is a known adverse effect of the drug and includes photosensitivity, keratinocyte proliferation and differentiation dysfunctions; the development of malignant lesions of the skin has also been reported (11).

The immediate progression of the disease noted after discontinuation of the drug and the prompt response after Vemurafenib reintroduction prove the strong dependence of the disease on the continuous inhibition of the BRAF^V600E^ activity. This evidence underlines the need for further combinations/options to possibly achieve complete tumor eradication.

The efficacy of the treatment in our small cohort is promising, with approximately 60% of response, although the small number of patients, given by the rarity of the condition, does not allow to draw firm conclusions on a statistically significant base. Only one patient with a bulky neurocytoma of the brainstem did not respond to the treatment (12). Taking into consideration the tolerability of the treatment, it represents a valuable option for these patients, sparing the long-term neurocognitive and endocrinological sequelae associated with chemotherapy and radiotherapy in a group of patients that have an excellent overall survival. Therefore, it is extremely important to reduce the possible impact of the treatment for these children and to preserve their quality of life during adulthood. It must be, however, underlined that evidence of the long-term safety of the treatment with Vemurafenib is lacking and should be prospectively confirmed. Moreover, as mentioned, although it can represent an excellent option to control the disease and could serve as a bridge to a more definitive treatment, neither it can be considered as a definitively curative approach itself nor a lifelong treatment can be envisioned.

Moreover, the regrowth of the disease observed under treatment, suggests the development of resistance, a well-documented finding in melanoma. The biological mechanism underlying the development of resistance has not been unraveled yet, but several studies are currently ongoing to find novel therapeutic strategies for these patients (13–15). Further molecular characterizations of the non-responders is necessary to evaluate the alternative pathways of the MAPK signaling.

Confirmation of our findings in larger and prospective studies is required. However, our results suggest that Vemurafenib could be a well-tolerated and effective option in pediatric patients affected by *BRAF* V600E-mutated LGG.

## Author contributions

FD, AM, and FL designed the study. FD, GC, AC, and IA cured the collection of the data. FD, GC, IA, AC, AM, GSC, ED FD, and F-DC interpreted and analyzed the data, FD and GC drafted the manuscript, AM and FL critically revised the manuscript for intellectual content. EA took care of the molecular characterization of the BRAF gene.

### Conflict of interest statement

The authors declare that the research was conducted in the absence of any commercial or financial relationships that could be construed as a potential conflict of interest.
